# Protectin conjugates in tissue regeneration 1 alleviates sepsis-induced acute lung injury by inhibiting ferroptosis

**DOI:** 10.1186/s12967-023-04111-9

**Published:** 2023-04-30

**Authors:** Ya Lv, Deming Chen, Xinyi Tian, Ji Xiao, Congcong Xu, Linan Du, Jiacong Li, Siyu Zhou, Yuxiang Chen, Rong Zhuang, Yuqiang Gong, Binyu Ying, Fang Gao-Smith, Shengwei Jin, Ye Gao

**Affiliations:** 1 Department of Anaesthesia and Critical Care, The Second Affiliated Hospital and Yuying Children’s Hospital of Wenzhou Medical University, Wenzhou, Zhejiang China; 2 Key Laboratory of Anesthesiology of Zhejiang Province, The Second Affiliated Hospital and Yuying Children’s Hospital of Wenzhou Medical University, Zhejiang, China; 3 Birmingham Acute Care Research Center, Institute of Inflammation and Ageing, University of Birmingham, Birmingham, UK; 4 Department of Anesthesiology, Hunan Cancer Hospital, No. 283, Tongzipo Road, Changsha, 410013 Hunan China; 5 The Second School of Medicine, Wenzhou Medical University, Wenzhou, Zhejiang China

**Keywords:** PCTR1, Ferroptosis, Sepsis, Acute lung injury, CREB

## Abstract

**Background:**

Acute lung injury (ALI) is a common and serious complication of sepsis with high mortality. Ferroptosis, categorized as programmed cell death, contributes to the development of lung injury. Protectin conjugates in tissue regeneration 1 (PCTR1) is an endogenous lipid mediator that exerts protective effects against multiorgan injury. However, the role of PCTR1 in the ferroptosis of sepsis-related ALI remains unknown.

**Methods:**

A pulmonary epithelial cell line and a mouse model of ALI stimulated with lipopolysaccharide (LPS) were established in vitro and in vivo. Ferroptosis biomarkers, including ferrous (Fe^2+^), glutathione (GSH), malondialdehyde (MDA) and 4-Hydroxynonenal (4-HNE), were assessed by relevant assay kits. Glutathione peroxidase 4 (GPX4) and prostaglandin-endoperoxide synthase 2 (PTGS2) protein levels were determined by western blotting. Lipid peroxides were examined by fluorescence microscopy and flow cytometry. Cell viability was determined by a CCK-8 assay kit. The ultrastructure of mitochondria was observed with transmission electron microscopy. Morphology and inflammatory cytokine levels predicted the severity of lung injury. Afterward, related inhibitors were used to explore the potential mechanism by which PCTR1 regulates ferroptosis.

**Results:**

PCTR1 treatment protected mice from LPS-induced lung injury, which was consistent with the effect of the ferroptosis inhibitor ferrostatin-1. PCTR1 treatment decreased Fe^2+^, PTGS2 and lipid reactive oxygen species (ROS) contents, increased GSH and GPX4 levels and ameliorated mitochondrial ultrastructural injury. Administration of LPS or the ferroptosis agonist RSL3 resulted in reduced cell viability, which was rescued by PCTR1. Mechanistically, inhibition of the PCTR1 receptor lipoxin A4 (ALX), protein kinase A (PKA) and transcription factor cAMP-response element binding protein (CREB) partly decreased PCTR1 upregulated GPX4 expression and a CREB inhibitor blocked the effects ofPCTR1 on ferroptosis inhibition and lung protection.

**Conclusion:**

This study suggests that PCTR1 suppresses LPS-induced ferroptosis via the ALX/PKA/CREB signaling pathway, which may offer promising therapeutic prospects in sepsis-related ALI.

**Supplementary Information:**

The online version contains supplementary material available at 10.1186/s12967-023-04111-9.

## Introduction

Sepsis, a life-threatening systemic illness, is primarily responsible for hospital mortality, and is also the dominant cause of ALI [1, 2]. Previous studies have demonstrated that inflammation, coagulation dysfunction and oxidative stress play significant roles in the development of sepsis-associated ALI, which manifests as massive inflammatory cell infiltration, progressive pulmonary filling, intractable arterial hypoxemia and dysfunction of lung tissue [3, 4]. However, the pathological mechanism of sepsis-induced ALI is still not fully understood. Notably, recent studies have indicated that ferroptosis plays an important role in the pathogenesis of sepsis-induced ALI.

Ferroptosis, which is different from apoptosis, autophagy and other forms of cell death, is driven by iron-dependent lipid peroxidation. Biochemically, the mechanisms underlying ferroptosis mainly include intracellular iron storage, GSH depletion, GPX4 inactivation and lipid peroxidation [5–8]. During ferroptosis in ALI, the production of lipid ROS induced by iron overload and dysfunction of the antioxidant system lead to the destruction of pulmonary epithelial cells and pulmonary vascular endothelial cells [9, 10]. In addition, ferroptosis has attracted tremendous interest for its unique correlation with a number of organ injuries during sepsis, including cardiovascular dysfunction, liver injury and kidney injury [11–14]. Hence, targeting ferroptosis is a possible therapeutic option for sepsis-induced multiple-organ dysfunction.

PCTR1, a new member of the specialized proresolving mediators (SPMs), is derived from docosahexaenoic acid (DHA) in leukocytes and highly abundant in lymphatic tissues [15]. PCTR1 not only promotes macrophage phagocytosis and efferocytosis to facilitate inflammation resolution but also accelerates tissue repair [16, 17]. Our previous study found that PCTR1 increased serum superoxide dismutase (SOD) and GPX4 levels and reduced ROS production in mice, thus ameliorating sepsis-related multiple organ damage [18]. Nevertheless, a connection between PCTR1 and ferroptosis has not been discovered.

CREB protein which is localized in the nucleus, binds to the cAMP response element (CRE) on the promoters of the target genes, once it has been phosphorylated at Ser133 by different protein kinases, including mitogen-activated protein kinases (MAPK), calmodulin-dependent protein kinase (CaMK), PKA and other kinases [19]. Phosphorylation of CREB has been shown to be protective in lung injury models, such as mediating transcription of VE-cadherin to promote lung vascular integrity and enhancing ENaC expression to remove alveolar fluid [20, 21]. Recently, the transcription factor CREB was identified to bind to the − 250 to − 1 region of the GPX4 promoter in H1299 and A549 lung cancer cells to inhibit ferroptosis [22].

In this study, we aimed to investigate the role of PCTR1 in the regulation of ferroptosis in sepsis-induced ALI and the potential mechanism, which mainly focuses on the CREB-associated regulation of ferroptosis.

## Materials and methods

### Reagents

PCTR1 (16-glutathionyl,17-hydroxy-4Z,7Z,10,12,14,19Z-docosahexaenoic acid) and H89 (PKA antagonist) were purchased from Cayman Chemical (Ann Arbor, MI). LPS (*Escherichia coli* 055: B5) and ferrostatin-1(ferroptosis antagonist) were purchased from Sigma (St. Louis, MO, USA). 1S,3R-RSL3 (ferroptosis agonist), 666-15 (CREB antagonist), MCC950 (pyroptosis antagonist) and Necrostatin-1 (necroptosis antagonist) were obtained from Med Chem Express (New Jersey, USA). BOC-2 (ALX receptor inhibitor) was purchased from Biomol-Enzo Life Sciences (Farmingdale, NY).

### Animal preparation

Male C57BL/6 mice (8–12 weeks old, 22–25 g), were purchased from Shanghai Experimental Animal Center of China. Mice were housed in specific pathogen-free rooms, which maintained a light/dark cycle for 12 h with controlled air temperature (22–26 °C) and relative humidity (60–65%). Mice had free access to water and food. This study was authorized by the Animal Studies Ethics Committee of Wenzhou Medical University and was implemented in accordance with the Guide for the Care and Use of Laboratory Animals. The sepsis-associated acute lung injury model was established by intraperitoneal injection of LPS (15 mg/kg) (or an equal volume of saline for the control group) according to our previous experiments [23].

### Cell culture

H1299 cells (human NSCLC cell line) were purchased from ATCC (Manassas, Virginia, USA). H1299 cells were cultured in RPMI 1640 medium supplemented with 10% fetal bovine serum and 1% penicillin‒streptomycin solution. Cells were cultured in an incubator at a constant temperature of 37 °C and 5% carbon dioxide. Cells were passaged when they reached 70–80% confluency. The third to fifth generation H1299 cells were used for the experiments.

### Cell viability determination

Cell viability was assessed by using Cell Counting Kit-8 (CCK-8, Dojindo, Tokyo, Japan) according to the manufacturer’s protocol. H1299 cells were seeded in 96-well plates at a density of 5000 cells/well and incubated for 24 h prior to various treatments. At the indicated time, 10 μL CCK-8 solution was added to each well, and the cells were incubated for another 3 h at 37 °C. The optical density (OD) values at 450 nm were measured using a microplate spectrophotometer.

### Western blotting

Lung tissues and H1299 cell proteins were extracted with lysis buffer. The total protein concentrations were then quantified with a BCA protein assay kit (Rockford, IL, USA). Equal amounts of protein (30 mg) from each group were loaded onto 10–12% SDS‒PAGE gels and then transferred to PVDF membranes. After being blocked with quick blocking buffer for 30 min, the membranes were incubated with primary antibodies against GPX4 (1:1000, BOSTER, Wuhan, China), PTGS2 (1:1000, Abcam, Cambridge, MA, USA), PKA (1:1000, Abcam, Cambridge, MA, USA), p-PKA(1:1000, Abcam, Cambridge, MA, USA), CREB(1:1000, Abcam, Cambridge, MA, USA), p-CREB (1:1000, Abcam, Cambridge, MA, USA), and β-actin (1:1000, BOYUN, Shanghai, China) overnight at 4 °C. After being rinsed with PBS three times, the membranes were incubated with secondary antibodies (1:2000, Santa Cruz Company) at room temperature for 1 h. The protein bands were detected by an Image Quant LAS 4000 mini (GE Healthcare Bio-Sciences AB, Uppsala, Sweden) and the band intensities were evaluated with ImageJ.

### Quantitative real-time PCR

Total RNA in lung tissues was extracted using TRIzol reagent purchased from Invitrogen (Carlsbad, CA, United States). cDNAs were reverse transcribed from mRNA by a reverse transcription kit in accordance with the manufacturer’s instructions (Thermo, Rockford, IL, USA). Gene expression was evaluated using the TB Green System (TaKaRa, Japan). Gene expression levels were normalized to the housekeeping gene β-actin. Data were analyzed by using the 2-ΔΔCt method. The gene-specific primers are summarized below.

TNF-α-F 5′-CCCTCACACTCACAAACCAC-3′ and TNF-α-R 5′-ACAAGGTACAACCCATCGGC-3′;

IL-1β-F 5′-ACAGCAGCATCTCGACAAGAGC-3′ and IL-1β-R 5′-CCACGGGCAAGACATAGGTAGC-3′;

LI-6-F 5′-TGCCACCTTTTGACAGTGATG-3′ and LI-6-R 5′-TGATGTGCTGCTGCGAGATT-3′;β-actin-F 5′-ACCCTAAGGCCAACCGTGAA-3′ and β-actin-R 5′-ATGGCGTGAGGGAGAGCATAG-3′.

### MDA, GSH, 4-HNE and Fe^2+^ measurements

GSH, MDA, 4HNE and Fe^2+^ in lung tissues and cells were respectively measured using a reduced GSH assay kit (A006-2-1), a MDA test kit (A003-1), a 4-HNE ELISA kit (H268) and an iron assay kit (A039-2-1) purchased from Nanjing Jiancheng Bioengineering Institute (Nanjing, China), in accordance with the manufacturer’s instructions.

### Lipid ROS detection

Cellular lipid ROS were determined using the C11 BODIPY 581/591 molecular probe (Invitrogen, Carlsbad, CA, USA). H1299 cells with different treatments were washed with PBS three times and then incubated with 1 mL of medium containing 10 μM C11 BODIPY 581/591 reagent for an additional 1 h. At the end time point, H1299 cells were washed with PBS three times, 1 mL of medium was added, and the cells were finally observed using an inverted fluorescence microscope (Nikon Eclipse Ts2). For quantitative analyses of lipid ROS in cells, flow cytometry was used. The cells were collected, washed three times with PBS, and then suspended in 1 mL PBS. The oxidized C11-BODIPY 581/591 probe was assessed with a Cytoflex (Beckman Coulter) and the data were analyzed by CytExpert 2.4 software.

### Pulmonary histopathological pathologic analysis

Left lungs were fixed with 4% paraformaldehyde at room temperature for 24 h, dehydrated, embedded in paraffin, stained using hematoxylin and eosin (H&E), and finally observed with a light microscope. The lung injury scores were quantified by two observers blinded to the treatment of each group according to the applicable histopathological scoring system.

### Immunofluorescence staining

H1299 cells were fixed in 4% paraformaldehyde for 15 min, permeabilized for 20 min with 0.1% Triton X-100, and blocked for 30 min with 10% donkey serum. Cells were incubated in anti-GPX4 (1:200) overnight at 4 °C and then incubated with secondary antibody for 1 h at 37 °C. The cell nucleus was counterstained with DAPI. Finally, fluorescence microscopy (Leica) was used to capture the cell images.

### Transmission electron microscopy

Lung tissue samples were prefixed in 2.5% glutaraldehyde for 24 h and postfixed in 1% osmium tetroxide for 1 h. After three washes with PBS, the specimens were sequentially dehydrated in gradient acetone followed by embedding in epoxy resin. The samples were dyed with uranyl acetate and lead citrate, and then cut into 50–70 nm ultrathin sections. Subsequently, images were obtained by a Zeiss EM 10C transmission electron microscope (Hitachi, H-7500, Tokyo, Japan).

### Statistical analysis

The Shapiro–Wilk test was used to test the normality of the data. All normally distributed data are presented as the means ± SD. For comparisons among multiple groups, the data were analyzed using one-way ANOVA, followed by Tukey’s post hoc test. For comparisons between two groups, an unpaired Student’s t test was performed. For non-normally distributed data (acute lung injury scores), the data are shown as the median and range (25th–75th percentile). The Kruskal–Wallis test was employed to determine the differences in the acute lung injury scores, and multiple comparisons were derived from the Mann‐Whitney U test. Statistical analyses were performed using GraphPad Prism software (version 8.0.1). Statistical significance was set at *p* < 0.05.

## Results

### Ferroptosis is induced in LPS-challenged ALI and associated with lung injury

**Fig. 1:**
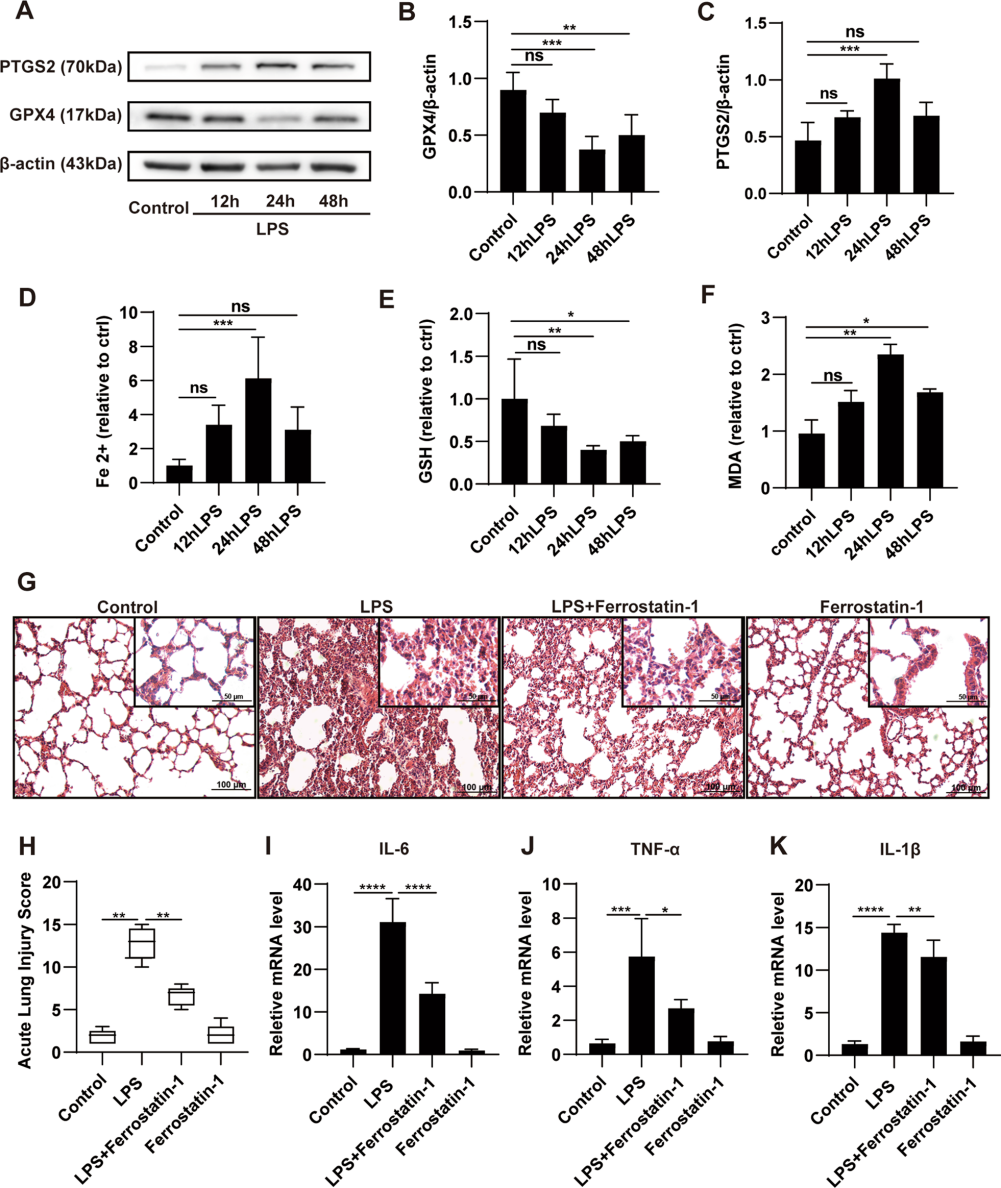
Ferroptosis is induced in LPS-induced ALI and associated with lung damage. LPS (15 mg/kg) in saline or an equivalent volume of saline was intraperitoneally injected into mice, and lung tissues were collected at 0, 12, 24, and 48 h, respectively. **A–C** Representative western blotting and quantification analysis of GPX4 and PTGS2. **D–F** Relative values of Fe^2+^, GSH and MDA concentrations. Mice were pretreated with ferrostatin-1 (10 mg/kg, ip) 1h1 h before being injected with LPS (15 mg/kg, ip). Lung samples were collected 24 h after LPS injection. **G** Representative H&E staining of lung tissues (original magnification, ×200; inset, ×400). **H** Acute lung injury score of each group. **I–K** The relative mRNA expression levels of the inflammatory cytokines: IL-6, TNF-α and IL-1β. The acute lung injury score data are presented as the median and range (25th–75th percentile), and other data are presented as the mean ± SD. n = 4–6. *p < 0.05, **p < 0.01, ***p < 0.001, ****p < 0.0001 and ns: *p* > 0.05. ip: intraperitoneal

The mouse model of sepsis-induced ALI was established by intraperitoneal (i.p.) injection with 15 mg/kg LPS. To explore the dynamic changes in ferroptosis in ALI, mice were exposed to LPS for 0, 12, 24 and 48 h. GPX4 and PTGS2 are prominent ferroptosis biomarkers. As shown in Fig. 1A–C, the downregulation of GPX4 and upregulation of PTGS2 were consistently induced by LPS, which were more obvious at 24 h than at 12 h or 48 h. Fe^2+^ and lipid peroxidation MDA also peaked at 24 h, accompanied by a remarkable decrease in GSH (Fig. 1D–F). Therefore, we chose 24 h as the stimulation duration of LPS in the subsequent experiments. Next, the ferroptosis inhibitor ferrostatin-1 was used to verify the effect of ferroptosis resistance on LPS-induced lung injury. As illustrated in Fig. 1G, H, compared with the control group, LPS stimulation dramatically led to pathological structural damage in the lungs, as indicated by increased acute lung injury scores, which determined from hemorrhage, alveolar edema, and inflammatory cell infiltration. However, treatment with ferrostatin-1 effectively ameliorated these changes as evidenced by decreased lung injury scores. Furthermore, the relative mRNA levels of proinflammatory cytokines, including IL-1β, IL-6 and TNF-α, were markedly increased in septic mice relative to normal mice, whereas these levels were distinctly decreased by ferrostatin-1 treatment (Fig. 1I–K). These results suggest that ferroptosis is implicated in the process of ALI development and that suppressing ferroptosis can reduce lung damage to some extent.

### PCTR1 alleviates lung injury by inhibiting ferroptosis

**Fig. 2:**
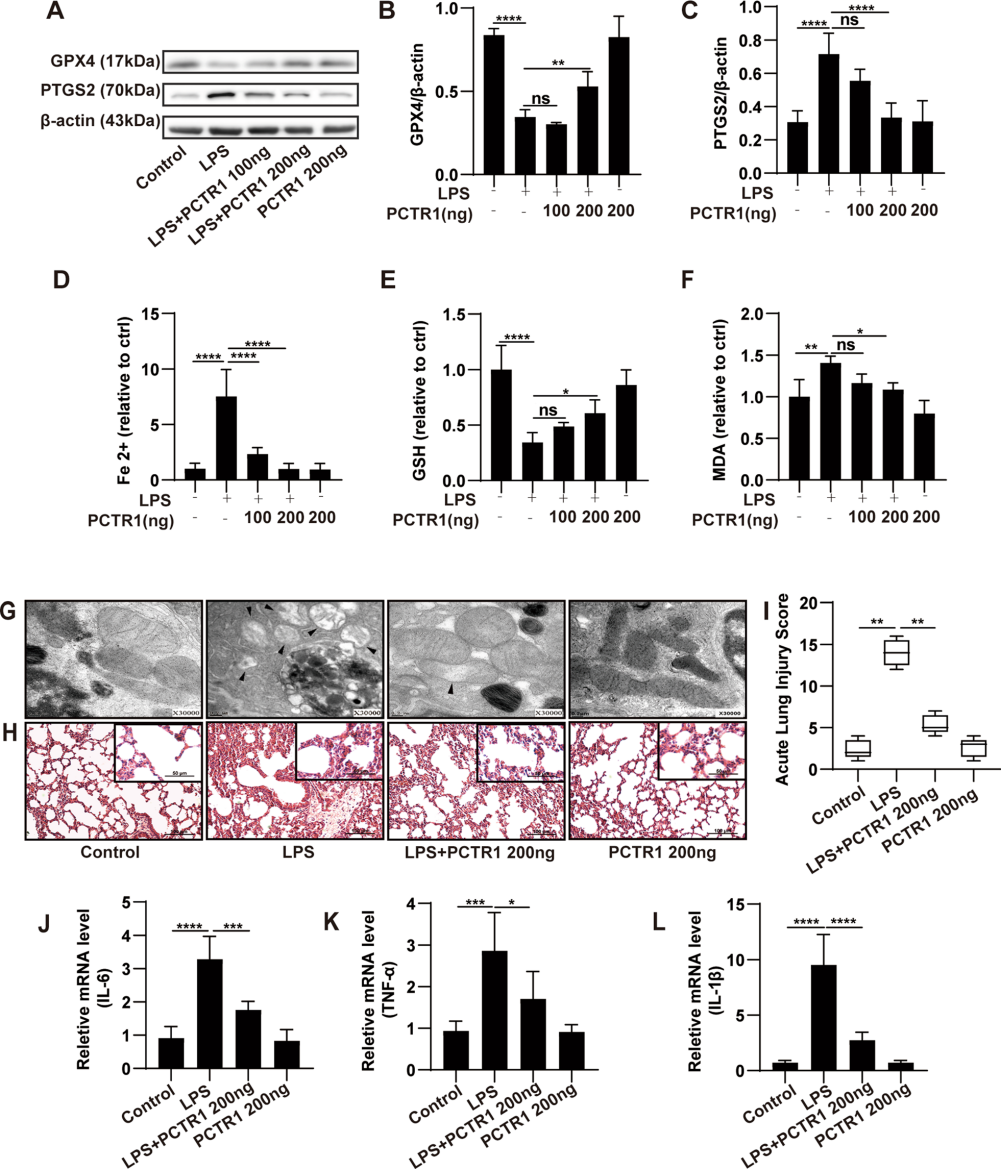
Effects of PCTR1 on ferroptosis in LPS-induced ALI. PCTR1 (100 or 200 ng) was injected into the caudal vein of mice 6 h after LPS (15 mg/kg, ip) treatment. All lung specimens were harvested at 24 h after LPS stimulation. **A–C** Representative western blotting and quantification analysis of GPX4 and PTGS2. **D–F** Relative values of Fe^2+^, GSH and MDA concentrations. **G** Representative TEM images of each group. The black arrow indicates ferroptotic mitochondria. Magnification ×30,000. **H** Representative H&E staining of lung tissues (original magnification, ×200; inset, ×400). **I** Acute lung injury score. **J–L** The relative mRNA expression levels of the inflammatory cytokines: IL-6, TNF-α and IL-1β. The acute lung injury score data are presented as the median and range (25th–75th percentile), and other data are presented as the mean ± SD. n = 4–6. *p < 0.05, **p < 0.01, ***p < 0.001, ****p < 0.0001 and ns: *p* > 0.05. ip: intraperitoneal

We then aimed to investigate the actions of PCTR1 on ferroptosis in LPS-induced ALI in vivo. First, after administration of LPS, PCTR1 (100 ng or 200 ng per mouse) was intravenously injected into mice to evaluate the effect of PCTR1 on ferroptosis. Although 100 ng PCTR1 treatment decreased the LPS-stimulated Fe^2+^ levels, there were no significant differences in the levels of GPX4, PTGS2, GSH and MDA (Fig. 2A–F). Compared with the LPS group, PCTR1 at 200 ng markedly promoted GPX4 expression and inhibited PTGS2 expression (Fig. 2A–C). Additionally, LPS-induced increase in MDA and Fe^2+^ and decrease in GSH were also reversed by 200 ng PCTR1. Consequently, PCTR1 at a concentration of 200 ng was selected in our follow-up experiments. Mitochondrial morphology is important for characterizing ferroptosis. In Fig. 2G, a transmission electron microscope was used to observe the ultrastructure of mitochondria. LPS challenge induced mitochondria shrinkage, an increase in the mitochondrial bilayer membrane density and the disappearance of mitochondria cristae, which was reversed by RCTR1 treatment. We further tested the effect of PCTR1 on LPS-induced ALI. As shown in the HE staining, after LPS injection, the lung tissues displayed significant interstitial edema, pulmonary architecture destruction, hemorrhaging, and inflammatory cell infiltration. However, these pathological changes in the lungs were obviously reversed by PCTR1 treatment. The quantification of acute lung injury agreed with these pathological changes (Fig. 2H, I). Additionally, PCTR1 treatment markedly diminished the mRNA expression of the proinflammatory cytokines IL-1β, IL-6 and TNF-α in lung tissues, which were obviously increased with LPS stimulation (Fig. 2J–L). Thus, these data imply that PCTR1 suppresses ferroptosis and exerts protective effects on LPS-induced ALI.

### PCTR1 dampens ferroptosis by activating ALX/PKA/CREB in LPS-induced ALI

**Fig. 3:**
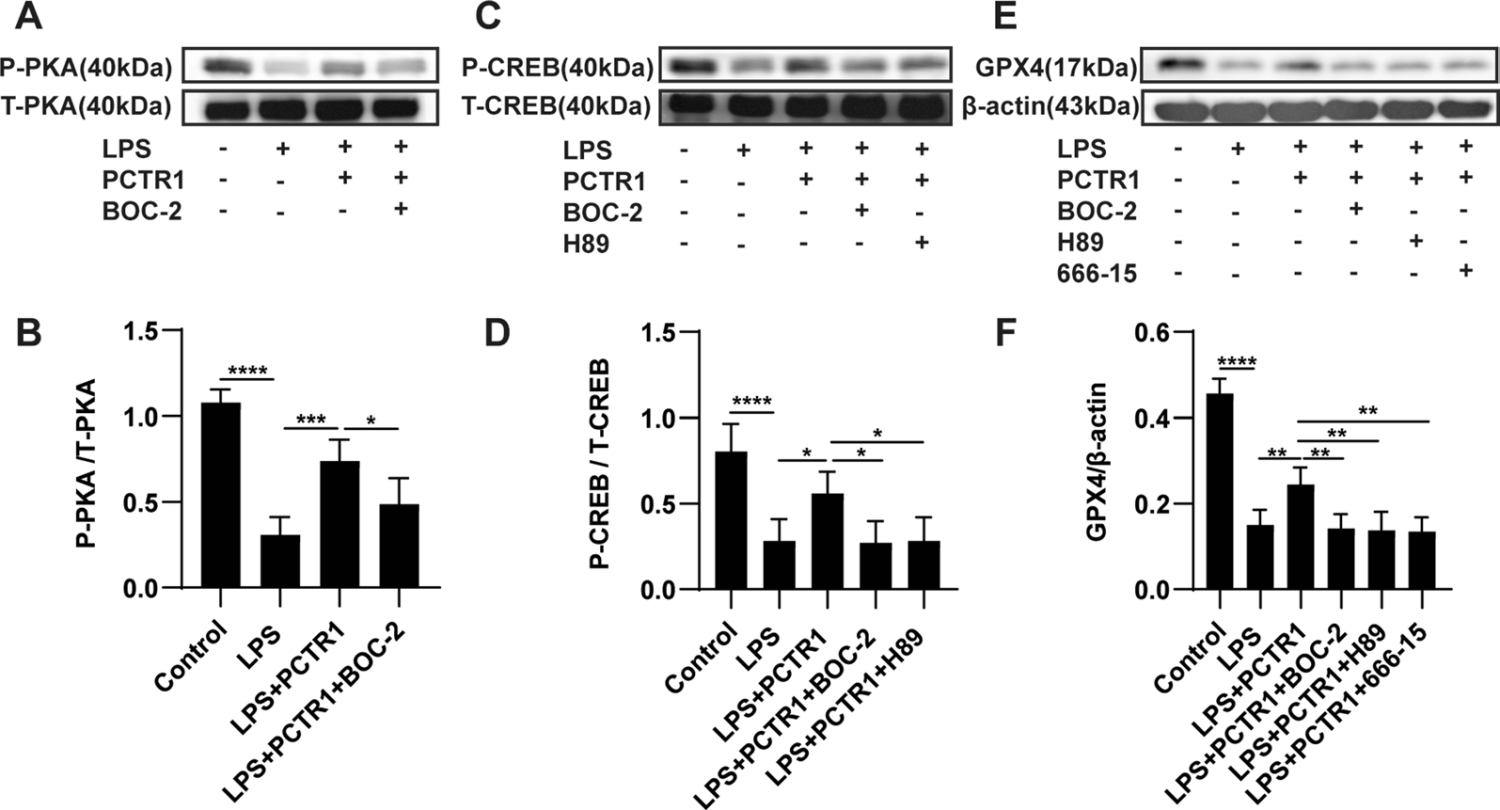
PCTR1 activates CREB via the ALX/PKA pathway to increase GPX4 expression in vivo. PCTR1 at a dose of 200 ng was injected into each mouse via the caudal vein 6 h after LPS (15 mg/kg, ip) administration. BOC‐2 (ALX receptor inhibitor, 600 ng/kg), H89 (PKA inhibitor, 10 mg/kg), 666-15 (CREB inhibitor, 10 mg/kg) or an equivalent volume of DMSO was injected into the caudal vein 1 h before PCTR1 treatment. The mice were sacrificed 24 h after LPS stimulation. **A**, **B** The protein expression level of P-PKA was determined by western blotting. **C**, **D** The protein expression level of P-CREB was determined by western blotting. **E**, **F** The protein expression level of GPX4 was determined by western blotting. Data are presented as the mean ± SD, n = 4–6. *p < 0.05, **p < 0.01, ***p < 0.001, ****p < 0.0001 and ns: *p* > 0.05. ip: intraperitoneal

**Fig. 4:**
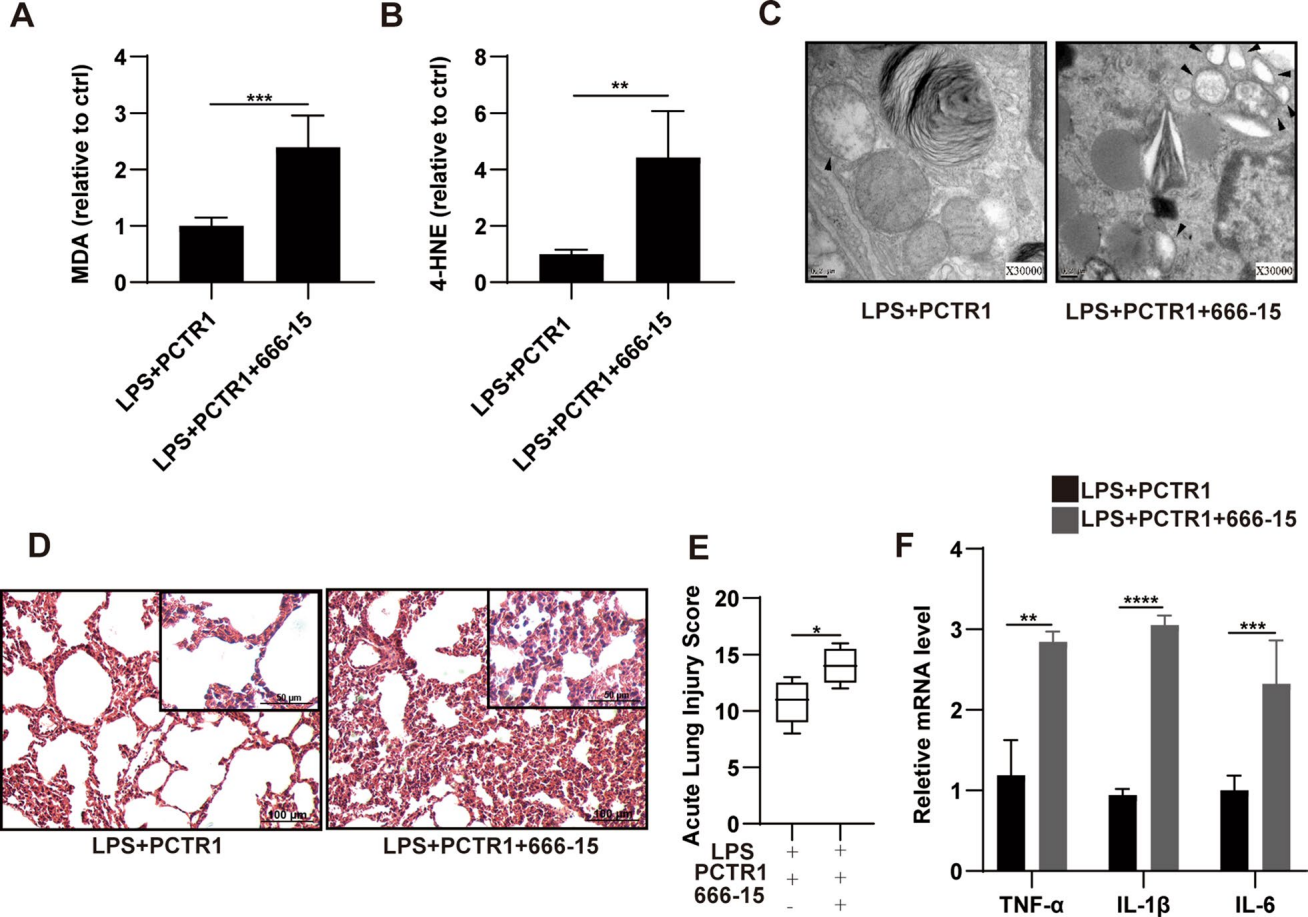
CREB mediates the effect of PCTR1 on eliminating lipid peroxides in LPS-induced ALI. PCTR1 at a dose of 200 ng was injected into each mouse via the caudal vein 6 h after LPS (15 mg/kg) administration. 666-15 (CREB inhibitor, 10 mg/kg) or an equivalent volume of DMSO was injected into the caudal vein 1 h before PCTR1 treatment. The mice were sacrificed 24 h after LPS stimulation. **A–B** Relative levels of MDA and 4-HNE. **C** Representative TEM images of each group. The black arrow indicates ferroptosis-like mitochondria. Magnification ×30,000. **D** Representative H&E staining of lung tissues (original magnification, ×200; inset, ×400). **E** Acute lung injury score. **F** The relative mRNA expression levels of the inflammatory cytokines: TNF-α, IL-1β and IL-6. The acute lung injury score data are presented as the median and range (25th–75th percentile), and other data are presented as the mean ± SD. n = 4–6. *p < 0.05, **p < 0.01, ***p < 0.001, ****p < 0.0001 and ns: *p* > 0.05

Subsequently, we explored the potential mechanism involved in the regulatory action of PCTR1 on ferroptosis in vivo. Our previous work reported that PCTR1 bound to the extracellular domains of its receptor ALX (a G-protein-coupled receptor) to exert its biological functions, which is critical for the activation of intracellular signaling cascades [18]. Protein kinase A (PKA), which was found to be a protein downstream of ALX in our previous research, phosphorylates CREB at Ser-133 [19, 24]. Phosphorylated CREB (p-CREB) can improve GPX4 expression at the transcriptional level [22]. Therefore, we explored whether PCTR1 activates CREB via the ALX/PKA pathway to upregulate GPX4 expression using specific inhibitors. After LPS stimulation, mice were pretreated with BOC-2 (ALX receptor inhibitor), H89 (PKA inhibitor), or 666-15 (CREB antagonist) before PCTR1 treatment. As shown in Fig. 3A, B, the phosphorylated PKA (p-PKA) level in the LPS group was obviously reduced compared with the control group, and PCTR1 treatment significantly enhanced the expression of p-PKA. However, the use of BOC-2 restrained the upregulation effect of PCTR1 on p-PKA, indicating that PCTR1 activates the ALX/PKA pathway. Next, we found that LPS-induced inhibition of p-CREB expression was dramatically released in the LPS + PCTR1 group, whereas BOC-2 or H89 treatment markedly inhibited PCTR1-induced phosphorylation of CREB (Fig. 3C, D). Finally, it was observed that BOC-2, H89, or 666-15 abolished the PCTR1-mediated upregulation of GPX4 (Fig. 3E, F). Collectively, these findings suggest that PCTR1 activates CREB by the ALX/PKA pathway to promote GPX4 expression. Since CREB could mediate PCTR1 to increase the expression of GPX4, we further verified whether CREB mediated the effect of PCTR1 on the elimination of lipid peroxides caused by GPX4 depletion in ferroptosis. Compared to the LPS + PCTR1 group, the CREB inhibitor 666-15 significantly increased MDA and 4-HNE levels (Fig. 4A, B). Typical ferroptotic mitochondria with reduced or absent cristae, a shrunken volume and increased membrane density were more abundant in the LPS + PCTR1 + 666-15 group (Fig. 4C). In addition, the lung tissues were more severely damaged and the inflammatory reaction was more intense due to the inhibition of CREB (Fig. 4D–F). These results suggest that PCTR1 likely dampens ferroptosis by activating ALX/PKA/CREB in LPS-induced ALI.

### PCTR1 inhibits LPS-induced ferroptosis in vitro

**Fig. 5:**
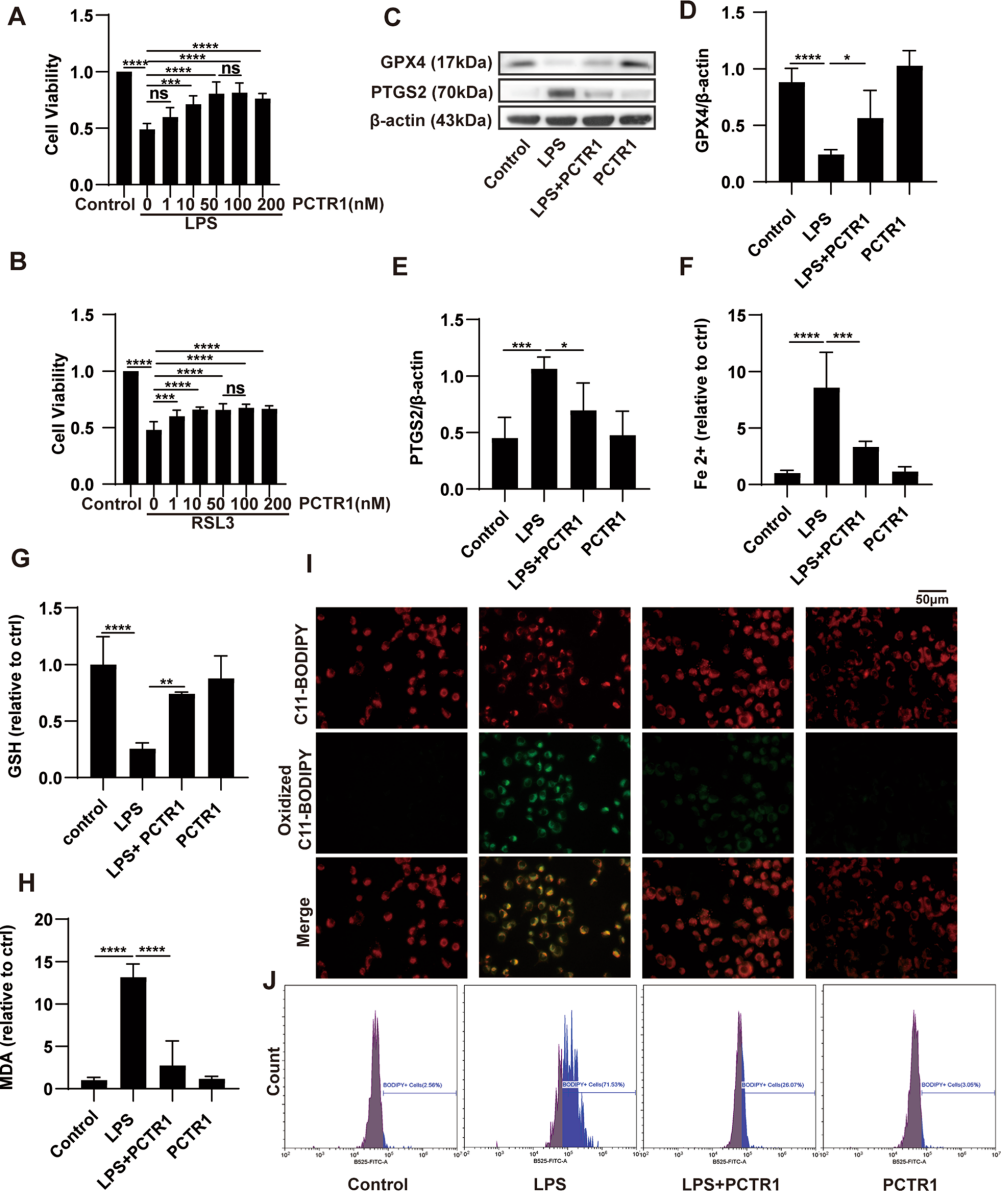
Effects of PCTR1 on LPS-induced ferroptosis in vitro. **A** H1299 cells were treated with LPS (10 μg/mL) and different concentrations of PCTR1 for 48 h. Fold change in cell viability. **B** H1299 cells were stimulated with RSL3 (10 nM) and different concentrations of PCTR1 for 48 h. Fold change in cell viability. **C–E** Representative western blotting and quantification analysis of GPX4 and PTGS2. **F–H** Relative levels of Fe^2+^, GSH and MDA. **I** The level of lipid peroxidation was determined with the C11-BODIPY 581/591 fluorescent probe (original magnification ×400). **J** Oxidized C11-BODIPY 581/591 probe was quantified by flow cytometry. Data are presented as the mean ± SD, n = 4–6. *p < 0.05, **p < 0.01, ***p < 0.001, ****p < 0.0001 and ns: *p* > 0.05

Alveolar epithelial cells, a critical component of the alveolar epithelial-endothelial barrier, play central roles in the pathogenesis of ALI. Ferroptosis was reported to participate in the injury of alveolar epithelial cells, which is induced by LPS [9]. Therefore, we next detect the effect of PCTR1 on LPS-induced ferroptosis in pulmonary epithelial cell line H1299. Cell viability was examined by the CCK-8 assay kit after H1299 cells were treated with different doses of LPS (0–1000 μg/mL) for 24 h. LPS did not induce the death of H1299 cells until its dose reached 50 μg/mL (Additional file 1: Fig. S1A). However, when the administration time of LPS was extended to 48 h, 5 or 10 μg/mL LPS was enough to reduce the cell viability statistically. There was a significant difference between 5 and 10 μg/mL (Additional file 1: Fig. S1B). Accordingly, we used 10 μg/mL LPS in cells for 48 h to establish a cell damage model in subsequent experiments. Moreover, ferrostatin-1, a specific ferroptosis inhibitor, was used to confirm that ferroptosis occurs in the decline of cell viability following LPS stimulation. As shown in Additional file 1: Fig. S1C, LPS- induced cell mortality was significantly abolished by ferrostatin-1 in a dose-dependent manner. Next, we also checked the levels of other cell death by using related antagonists. As shown in Additional file 1: Fig. S1D, compared with MCC950 (inhibitor of pyroptosis) and necrostatin-1 (inhibitor of necroptosis), the use of ferrostatin-1 most significantly improved the cell viability, indicating that ferroptosis is the major form of regulated cell death in our model. We then investigated the role of PCTR1 in LPS- induced ferroptosis in vitro. As illustrated in Fig. 5A, B, PCTR1 distinctly caused dose-dependent changes in cell viability when cotreatment with LPS or the ferroptosis agonist RSL3. When the concentration of PCTR1 was 50 or 100 nM, the cell viability increased most, and there was no significant difference between 50 and 100 nM. Therefore, we chose 50 nM of PCTR1 in the following experiment. Moreover, LPS challenge resulted in an obvious decrease in GPX4 and an increase in PTGS2, which were reversed by PCTR1 treatment (Fig. 5C–E). There was also a significant reduction in Fe^2+^ and MDA and an increase in GSH in the LPS + PCTR1 group compared with the LPS group (Fig. 5F–H). In addition, the production of oxidized C11 BODIPY 581/591, representing the level of lipid ROS, was observed by fluorescence microscopy and flow cytometry. Lipid ROS markedly accumulated in H1299 cells, which was captured by the increase in green fluorescence, while PCTR1 treatment significantly diminished the fluorescence intensity (Fig. 5I). Consistently, the inhibitory effect of PCTR1 on lipid ROS was also confirmed by flow cytometry (Fig. 5J). Taken together, these data demonstrate that PCTR1 protects alveolar epithelial cells against LPS-induced ferroptosis.

### PCTR1 suppresses ferroptosis by ALX/PKA/CREB activation in vitro

**Fig. 6:**
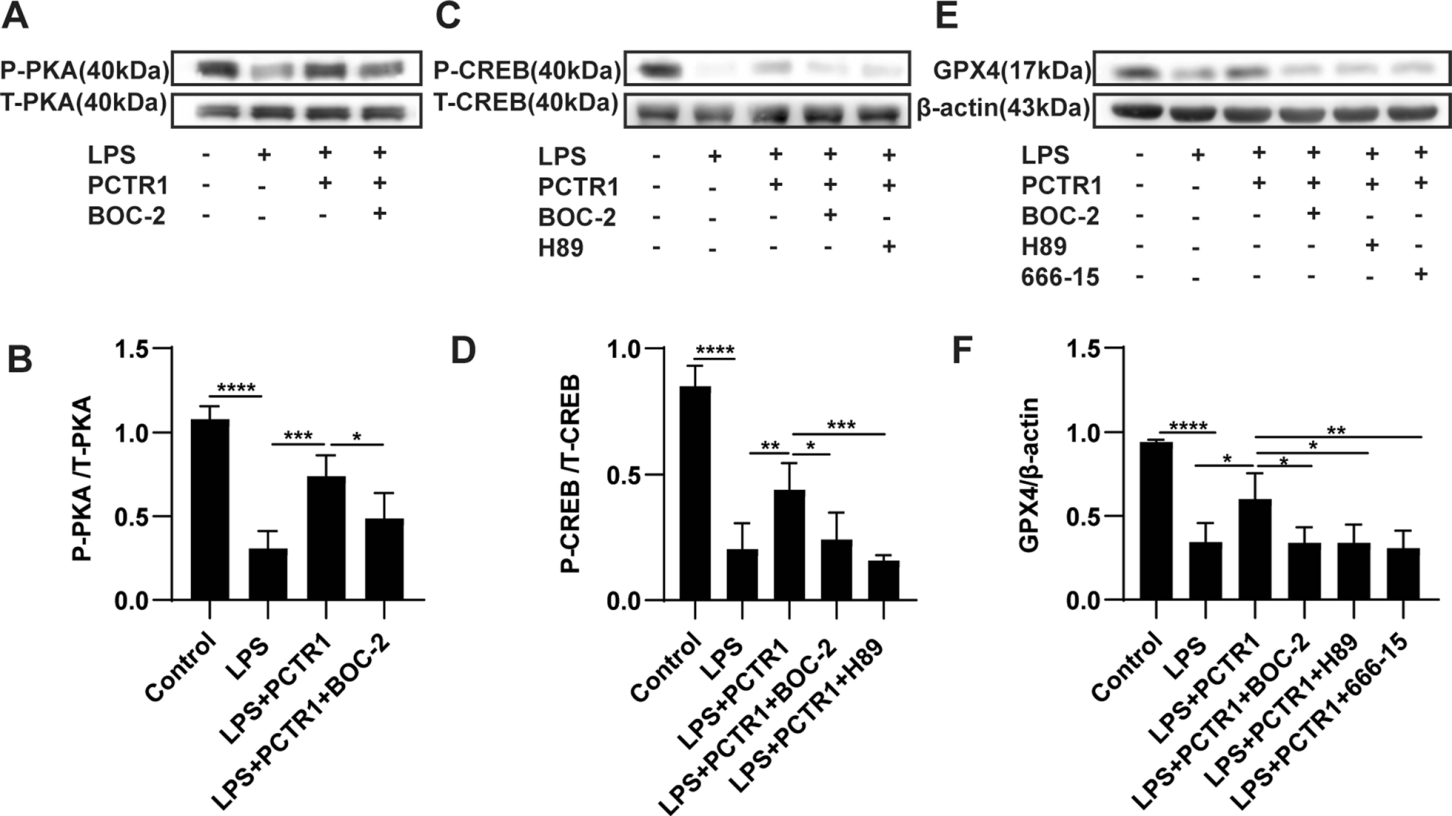
PCTR1 activates CREB by the ALX/PKA pathway to increase GPX4 expression in vitro. BOC-2 (ALX receptor inhibitor, 10 μM), H89 (PKA inhibitor, 10 μM), 666-15 (CREB inhibitor, 1 μM) or an equivalent volume of DMSO was administered to H1299 cells for 30 min in advance, and then LPS and PCTR1 were co-administered for 48 h. **A**, **B** The protein level of P- PKA was measured by western blot. **C**, **D** The protein level of P-CREB was measured by western blot. **E**, **F** The protein level of GPX4 was measured by western blot. Data are presented as the mean ± SD, n = 5–6. *p < 0.05, **p < 0.01, ***p < 0.001, ****p < 0.0001 and ns: *p* > 0.05

**Fig. 7:**
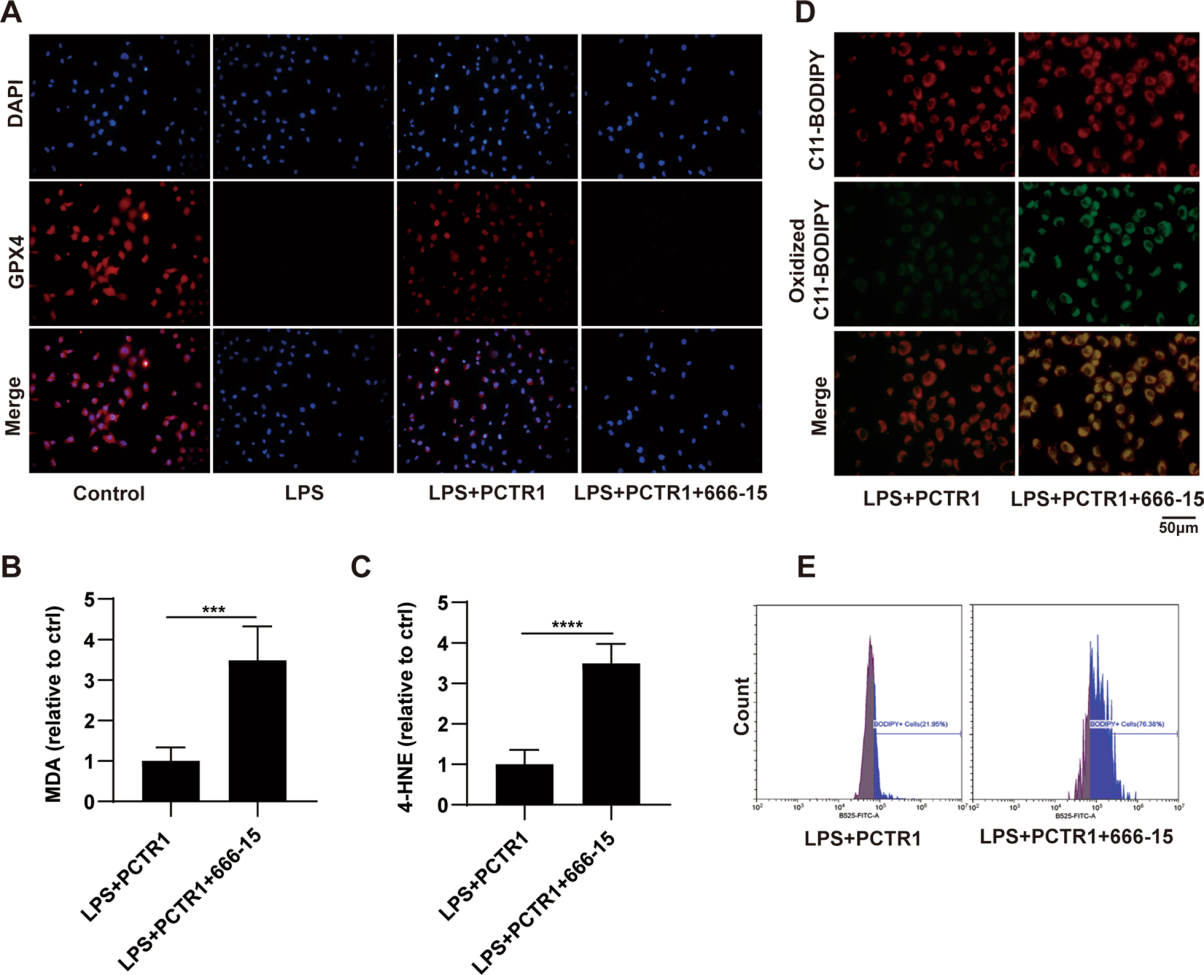
CREB mediates the effect of PCTR1 on eliminating lipid peroxides in vitro. 666-15 (CREB inhibitor, 1 μM) or an equivalent volume of DMSO was administered to H1299 cells for 30 min in advance, and then LPS and PCTR1 were co-administered for 48 h. **A** Immunofluorescence staining images of GPX4 (original magnification ×400). **B**, **C** Relative expression levels of GSH, MDA and 4-HNE. **D** The level of lipid peroxidation was determined with the C11-BODIPY 581/591 fluorescent probe (original magnification ×400). **E** Oxidized C11-BODIPY 581/591 probe was quantified by flow cytometry. Data are presented as the mean ± SD, n = 5–6. *p < 0.05, **p < 0.01, ***p < 0.001, ****p < 0.0001 and ns: *p* > 0.05

Similarly, we further explored whether PCTR1 phosphorylates CREB to elevate the expression of GPX4 via ALX/PKA in vitro. The protein level of p-PKA was obviously promoted in PCTR1-treated cells but diminished by cotreatment with BOC-2 (Fig. 6A, B). PCTR1 elevated the LPS-reduced expression of p-CREB, while the effect was partly abolished by BOC-2 or H89 administration (Fig. 6C, D). Moreover, PCTR1 enhanced LPS-induced decrease in GPX4 expression, whereas BOC-2, H89, or 666-15 reversed this decrease (Fig. 6E, F). These results indicate that PCTR1 activates CREB through the ALX/PKA pathway, thereby increasing the expression of GPX4. Next, we also investigated whether PCTR1 upregulated the expression of GPX4 and decreased accumulation of lipid peroxide via CREB in vitro. Immunofluorometric analysis demonstrated that GPX4 expression in the LPS + PCTR1 + 666-15 group was decreased compared with that in the LPS + PCTR1 group, which agreed with the western blotting results (Fig. 7A). The end products of lipid peroxidation of ferroptosis, such as MDA and 4-HNE, were enhanced when pretreatment with the CREB inhibitor 666-15 was performed before PCTR1 administration (Fig. 7B, C). In parallel, the results of the fluorescence microscopy and flow cytometry also indicated that inhibition of CREB with 666-15 attenuated the inhibitory effect of PCTR1 on LPS-induced lipid ROS (Fig. 7D, E). These results suggest that ALX/PKA/CREB signaling is responsible for the inhibitory effect of PCTR1 on LPS-induced ferroptosis.

## Discussion

**Fig. 8:**
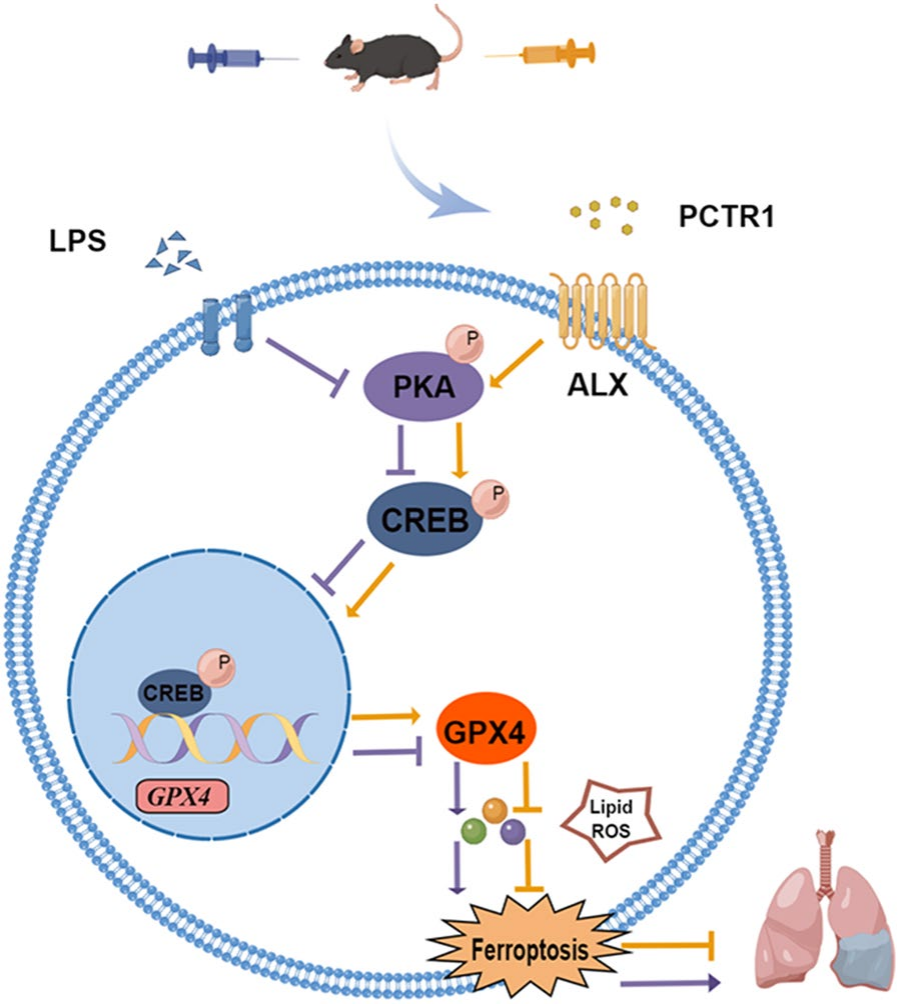
PCTR1 mitigates LPS-induced acute lung injury by inhibiting ferroptosis via ALX/PKA/CREB. The function of LPS is displayed in purple, while that of PCTR1 is shown in yellow

ALI is a serious respiratory disease correlated with dysfunction of the alveolar-capillary barrier caused by multiple factors [25, 26]. As the disease is related to high morbidity and mortality rates, it constitutes a primary public health burden [27, 28]. To date, its clinical treatments mainly contain protective mechanical ventilation, without effective drugs for its management [29, 30]. Therefore, it is important to elucidate its pathogenesis and develop effective drugs for its therapy. In the present study, we confirmed that PCTR1 could effectively ameliorate acute inflammation and morphological damage in LPS- stimulated lung injury mechanically by inhibiting ferroptosis. The use of antagonists BOC-2, H89, and 666-15 blocked the promoting effect of PCTR1 on GPX4 expression demonstrating that PCTR1 elevated the expression level of GPX4 by activating the ALX/PKA/CREB signaling pathway. The CREB inhibitor 666-15 partly abolished PCTR1-induced ferroptosis inhibition, implying that CREB was the key point in the regulation of PCTR1 on ferroptosis. Moreover, our in vitro experiments were also consistent with the findings in vivo studies. Taken together, our results indicate that PCTR1 alleviates LPS-induced ALI by suppressing ferroptosis, which depends on activating the ALX/PKA/CREB pathway (summarized in Fig. 8).

Ferroptosis is tightly controlled by redox equilibrium, iron homeostasis and lipid metabolism. Fe^2+^ can directly generate excessive ROS through fenton reaction and increase the activity of some enzymes responsible for oxygen homeostasis and lipid peroxidation [31]. In both in vivo and in vitro experiments, it was observed that the level of Fe^2+^ was increased upon LPS stimulation, but decreased with PCTR1 treatment. The cystine/glutamate antiporter system Xc− /GSH/GPX4 axis is a critical antioxidant system. The cystine/glutamate antiporter system Xc− sustains the production of GSH [32]. Two electrons offered mainly by GSH and the catalytic selenocysteine residue of GPX4 are required for GPX4 to reduce lipid peroxides (PL-OOH) to their corresponding alcohols (PL-OH) [33]. In the present study, GSH and GPX4 were markedly declined after LPS stimulation, but that was reversed by PCTR1. Lipid peroxidation is a free radical propelled reaction leading to the formation of various active compounds, including MDA and 4HNE, resulting in cellular damage [32]. We discovered that PCTR1 administration downregulated lipid peroxidation caused by LPS. In addition, treatment with RSL3, an inhibitor of GPX4 [34], induced a decline in cell viability, which was rescued by PCTR1 in vitro, suggesting the specific regulation of ferroptosis by PCTR1. In the morphological features of ferroptosis, changes in mitochondrial ultrastructure such as reduced or absent cristae, a shrunken volume and an increased membrane density are considered major events [32]. Mitochondria with those features were identified in our LPS-induced ALI model, while the mitochondrial structure was ameliorated after PCTR1 treatment. In addition, we found that LPS-induced inflammatory reactions and tissue damage were distinctly reduced by PCTR1, which is similar to the effect of the ferroptosis inhibitor ferrostatin-1. These findings provide evidence that PCTR1 might restore sepsis-related ALI by inhibiting ferroptosis.

PCTR1, a new member of the protectin family of specialized proresolving mediators, was reported to decrease classical proinflammatory mediators, exert a characteristic tissue regenerative role and enhance macrophage phagocytosis and efferocytosis [16, 17]. Our previous findings revealed that PCTR1 could protect against septic acute lung injury at least partly by promoting alveolar fluid clearance and restoring the lung vascular glycocalyx, ultimately improving survival rates [35, 36]. Moreover, we also found that PCTR1 improved serum SOD and GPX4 levels in septic mice with multiple-organ dysfunction [18], and therefore we speculated that PCTR1 might effectively inhibit ferroptosis by regulating GPX4. The Conrad group furnished early evidence that loss of GPX4 induced lipid peroxidation-dependent cell death in the embryonic fibroblasts of conditional GPX4 knockout mice [37]. A recent study further proposed that GPX4 plays an essential role in preventing lipid peroxidation-induced ferroptosis and acute renal failure [38]. Despite the prevailing importance of GPX4 for ferroptosis, little is known about how GPX4 is governed in ferroptosis in ALI. The majority of previous research has focused on nuclear factor-erythroid-2-related factor 2 (Nrf2), which translocates into the nucleus to elevate the expression level of GPX4, resulting in ferroptosis inhibition [39]. Knockout of Yes-associated protein 1 (YAP1), a key regulator of the Hippo signaling pathway, accelerated ferroptosis and exacerbated CLP-induced ALI by decreasing the expression of GPX4 [9]. Currently, several studies have proposed that CREB is involved in the regulation of GPX4 transcription, making CREB an important focus of ferroptosis research. Wang et al. have affirmed that CREB directly binds the promoter of GPX4 in H1299 cells via ChIP experiments [22]. Xiong et al. demonstrated that PM2.5 exposure induces ferroptosis in neuronal cells by inhibiting CREB [40]. Chen et al. indicated that the bioflavonoid galangin delivered its anti-ferroptosis effects by activating CREB in a hepatic ischemia‒reperfusion model [41]. Indeed, a link between CREB and GPX4 was previously found during enterocytic cell differentiation [42]. However, another recent study found the opposite effect of CREB on ferroptosis, revealing that CREB regulates acyl-CoA synthetase long-chain family member 4 (ACSL4) expression in hepatoma to promote ferroptosis and induce hepatotoxicity [43]. Therefore, the effect of CREB on ferroptosis might depend on the binding gene or the disease condition. In this study, we discovered the positive effect of CREB on ferroptosis inhibition in a sepsis-induced ALI model. In our experiments, we discovered that PCTR1 activates CREB by the ALX/PKA pathway to promote GPX4 expression by western blotting and immunofluorescence assays. Inhibition of CREB with 666-15 weakened the scavenging effect of PCTR1 on MDA and 4-HNE and hindered the ability of PCTR1 to reduce lung damage. Additionally, CREB negatively regulates other cell death modes, such as apoptosis and pyroptosis. Mice lacking CREB in the central nervous system show massive apoptosis of postmitotic neurons during development [44]. Transcriptional cooperation between CREB and Nrf2 enhances resistance to not only apoptosis but also pyroptosis, exerting an anti-inflammatory effect [45, 46].

Apart from regulating GPX4 to achieve redox balance, PCTR1, as a lipid metabolite, seems to have a complex relationship with lipid metabolism. Lipid metabolism is another key mechanism of ferroptosis. The peroxidation of polyunsaturated fatty acids (PUFAs) at the bis-allylic position is a critical step in promoting ferroptosis [47]. Arachidonic acid and adrenic acid (AA/AdA) are the core substrates of lipid peroxidation reactions in ferroptosis [48]. PUFAs are cleaved into free PUFAs and lysophospholipids by phospholipase A2 (PLA2). Only free AA/ADA can combine with Coenzyme A (CoA) to form AA/AdA-CoA, which subsequently reacts with membrane phosphatidylethanolamine (PE) to synthesize AA/AdA–PE. Interestingly, a previous study from our group showed that PCTR1 inhibits the translation of bound AA to free AA by inhibiting the expression of PLA2 [18]. Next, the mammalian arachidonic acid lipoxygenase (ALOX) family, including six members, mediates AA/AdA-PE to generate AA/AdA-PE-OOHs in a tissue- or cell-dependent manner, thus causing ferroptosis. For example, arachidonate-15-lipoxygenase (ALOX15) participates in TP53-induced ferroptosis in H1299 cells [49]. On the other hand, ALOX15 is also the key enzyme in the first step of PCTR1 production. First, ALOX15 catalyzes the production of 17S-hydroperoxy-docosahexae (17S-HpDHA) from DHA, then 17S-HpDHA produces 16S,17S-epoxy-protectin through an epoxidation reaction, and finally, PCTR1 is generated by human recombinant leukotriene C4 synthase (LTC4S) and glutathione S-transferases (GSTs) from 16S,17S-epoxy-protectin [50, 51]. Therefore, it is possible that the production of endogenous PCTR1 competes with the lipid peroxidation reaction because ALOX15 is required by both. We also hypothesized that exogenous administration of PCTR1 may negatively regulate ALOX15 enzymatic activity, thereby inhibiting the catalytic conversion of AA/AdA-PE to AA/ADA-PE-OOH. The complex crosstalk between PCTR1 and ferroptosis in terms of lipid metabolism awaits further investigation in our follow-up study.

In summary, our findings improve the understanding of the anti-ferroptosis effect mediated by PCTR1 and clarify the underlying mechanisms. The results suggest that PCTR1 might be a potentially effective drug for the treatment of ALI. There are also some limitations in the current study. ALI can be caused either by direct lung injury (e.g., pneumonia, aspiration) or indirect lung injury (e.g., endotoxemia, pancreatitis). LPS intraperitoneal administration induced-acute lung injury in this study is only a model of ALI. In addition, the differences in the pharmacokinetics of PCTR1 and immune functions between small animals and humans should also be considered.

## Conclusions

In conclusion, these data demonstrated that ferroptosis is implicated in the development of ALI and that PCTR1 potently protects against acute lung injury by inhibiting ferroptosis, which is mediated by ALX/PKA/CREB activation. This study enriches the regulatory mechanism of ferroptosis in sepsis-associated ALI and indicates that PCTR1 may become a therapeutic approach for ALI. Considering that ferroptosis is involved in the pathological process of many diseases and that PCTR1 effectively inhibits ferroptosis in our LPS-induced ALI model, we can further verify its therapeutic potential in the management of numerous ferroptosis-related diseases.

## Supplementary Information

**Additional file 1: Fig. S1.** Viability of H1299 cells stimulated by LPS at different concentrations for 24 h. Viability of H1299 cells stimulated by LPS at different concentrations for 48 h. H1299 cells were pretreated with or without different concentrations of ferrostatin-1 for 30 min, followed by LPSfor 48 h. H1299 cells were pretreated with ferrostatin-1, MCC950or Necrostatin-1for 30 min, followed by LPSfor 48 h. Fold change in cell viability. Data are presented as the mean ± SD, n = 5–6. *p < 0.05, **p < 0.01, ***p < 0.001, ****p < 0.0001 and ns: p > 0.05.

## Data Availability

The data that support the findings of this study are available from the corresponding author upon reasonable request. Some data may not be made available because of privacy or ethical restrictions.

## Abbreviations

ALI: Acute lung injury
AA: Arachidonic acid
AdA: Adrenic acid
ALOX15: Arachidonate-15-lipoxygenase
CREB: CAMP-response element binding protein
CRE: CAMP response element
CaMK: Calmodulin-dependent protein kinase
CoA: Coenzyme A
DHA: Docosahexaenoic acid
Fe^2+^: Ferrous
GSH: Glutathione
GPX4: Glutathione peroxidase 4
GSTs: Glutathione S-transferases
LPS: Lipopolysaccharides
Lipid ROS: Lipid reactive oxygen species
LTC4S: Leukotriene C4 synthase
MAPK: Mitogen-activated protein kinase
PCTR1: Protectin conjugates in tissue regeneration 1
PTGS2: Prostaglandin-endoperoxide synthase 2
PUFAs: Polyunsaturated fatty acids
PLA2: Phospholipase A2
PE: Phosphatidylethanolamine
PKA: Protein kinase A
SPMs: Specialized proresolving mediators
SOD: Serum superoxide dismutase
