# Supplementary figures and images for: Protectin conjugates in tissue regeneration 1 alleviates sepsis-induced acute lung injury by inhibiting ferroptosis
Source: J Transl Med. 2023 Apr 30;21:293. doi: 10.1186/s12967-023-04111-9 (PMC10150510; doi:10.1186/s12967-023-04111-9)

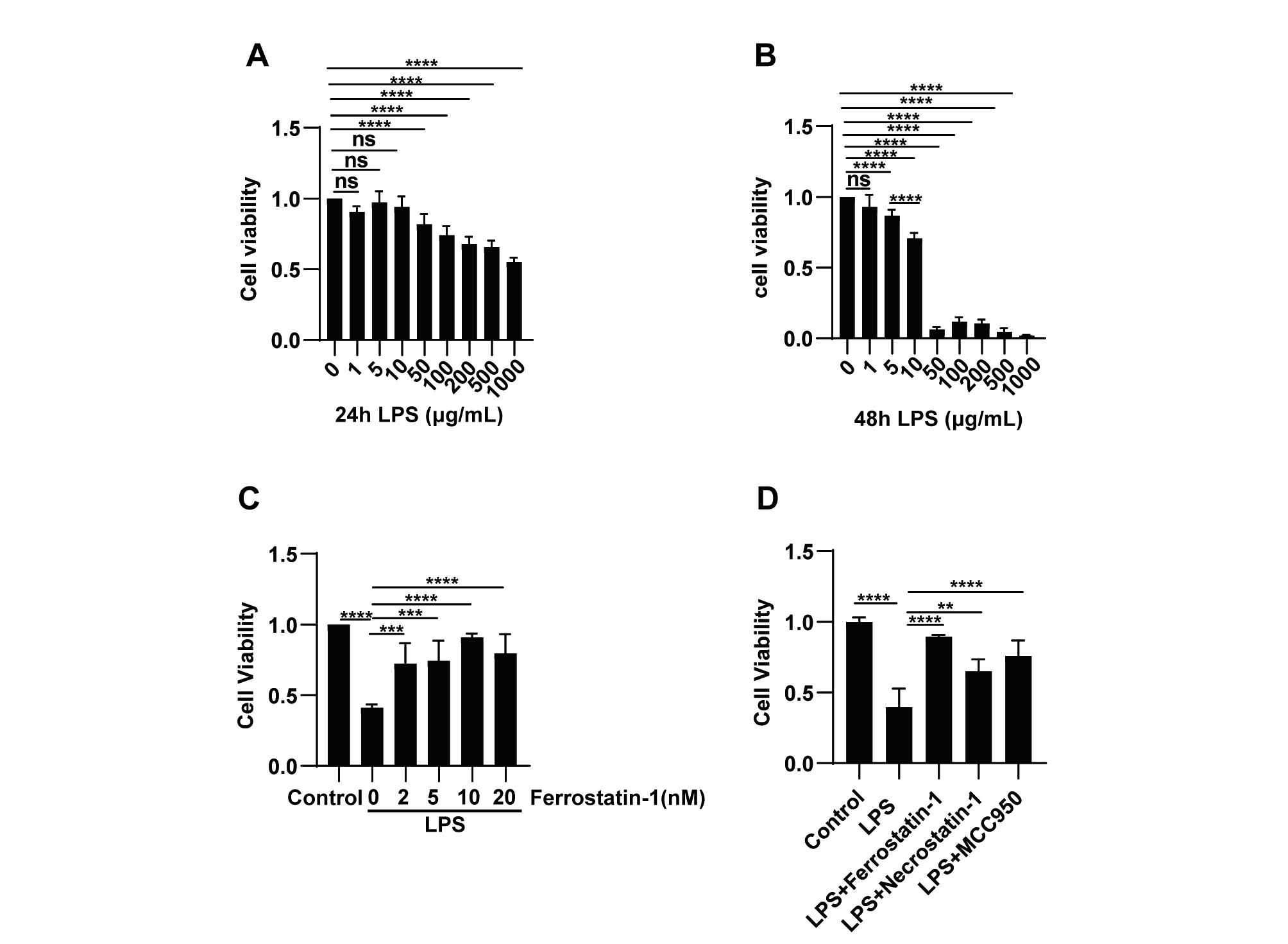

Supplement: Supplementary file 1 — Additional file 1: Fig. S1. Viability of H1299 cells stimulated by LPS at different concentrations for 24 h. Viability of H1299 cells stimulated by LPS at different concentrations for 48 h. H1299 cells were pretreated with or without different concentrations of ferrostatin-1 for 30 min, followed by LPSfor 48 h. H1299 cells were pretreated with ferrostatin-1, MCC950or Necrostatin-1for 30 min, followed by LPSfor 48 h. Fold change in cell viability. Data are presented as the mean ± SD, n = 5–6. *p < 0.05, **p < 0.01, ***p < 0.001, ****p < 0.0001 and ns: p > 0.05. [file 12967_2023_4111_MOESM1_ESM.tif]
